# Rural health and Family Medicine 

**DOI:** 10.4102/phcfm.v2i1.145

**Published:** 2010-05-24

**Authors:** Bernhard M. Gaede

**Affiliations:** 1 Department of Family Medicine, University of Pretoria, South Africa; 2 Emmaus Hospital, Winterton, South Africa

The recent successful 2nd Africa Regional WONCA (World Organisation of Family Doctors) Conference that was held in Rustenburg, South Africa, had the theme of the role of the family physician in Africa. A question of particular interest to me, being involved with, and advocating for, rural health, was the passionate discussion regarding extended procedural skills and whether these formed part of the core skills for family physicians in Africa.

The overall debate placed the family physician in the context of the current revival of primary health care (PHC) and the move from the hospital to the community. The underlying issue, however, is deeper than just being able to perform a certain procedure; it centres on the level of care available – in terms of rural health, this translates into whether family physicians should be functioning at the district hospital level. In rural areas, the scope of skills required at district hospitals includes the in-patient management of a wide range of conditions, many of which are life-threatening and severe. Specifically, the procedural skills needed in a remote area include being able to administer a safe anaesthetic and the required level of surgical skill with which to perform safe caesarean sections or appendectomies.

The resource deprivation, low population density and the limited number of beds in hospitals in rural areas make the appointment of specialists (obstetricians, surgeons, physicians or paediatricians) not a viable option for the vast majority of rural hospitals. Therefore, the in-patient care of people living in these areas becomes the responsibility of the district hospital generalist. The discussion at hand, though, should not focus on whether the specialists are needed, but on who will undertake the responsibility to guide and support the district hospital generalists and highlight the career options that are available to them, for, in rural areas, certainly, the generalists are here to stay. 

I would like to call on the community of Family Medicine to actively take on the leadership and  responsibility for rural health, including the training, support and guidance of district hospital generalist. This in no way contradicts the position of Family Medicine as focusing on PHC and moving toward community-oriented primary care (COPC), but rather allows for the potential creation of a continuum between the community, the clinic and the hospital. However, the implication in this is that Family Medicine departments in Africa will need to be able to train rural family physicians with thorough exposure in the range of skills needed to function at a district hospital. For rural health this is not a ‘nice-to-have’ option, but rather needs to be part of the core practices of Family Medicine.

The question of whether a single family physician would be able to cover the whole range of skills – COPC, primary clinical care, management, teaching and procedural skills – needs to be explored, as the scope and depth of these may vary from country to country. Creative approaches are needed to ensure the family physician does not lose sight of the fact that the hospital is not the most important site of action. However, solutions will arise as soon as it becomes clear that the district hospital generalist, indeed, has a home in Family Medicine.  

**FIGURE 1:**
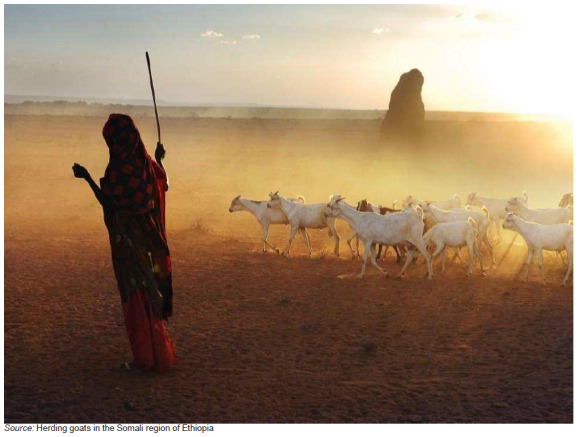
Source: Photo taken by Dieter Telemans. Herding goats in the Somali region of Ethiopia.

